# Metallic vs Ceramic Bracket Failures After 12 Months of Treatment: A Prospective Clinical Trial

**DOI:** 10.1016/j.identj.2024.04.023

**Published:** 2024-05-13

**Authors:** Andrea Scribante, Maurizio Pascadopoli, Paola Gandini, Riccardo Mangia, Costanza Spina, Maria Francesca Sfondrini

**Affiliations:** a Unit of Orthodontics and Pediatric Dentistry and Unit of Dental Hygiene, Section of Dentistry, Department of Clinical, Surgical, Diagnostic and Pediatric Sciences, University of Pavia, Pavia, Italy; b Unit of Orthodontics and Pediatric Dentistry, Section of Dentistry, Department of Clinical, Surgical, Diagnostic and Pediatric Sciences, University of Pavia, Pavia, Italy

**Keywords:** Orthodontic treatment, Fixed appliance, Metallic brackets, Ceramic brackets, Failure rate, Survival analysis, Clinical trial

## Abstract

**Introduction:**

Orthodontic treatment with fixed vestibular appliances is still widely used worldwide. When choosing the aesthetic alternative of ceramic brackets, the possibility of failure and cracking of braces should be considered. Therefore, the aim of the present study was to compare the failure rates of ceramic and metal brackets in a 12-month clinical study.

**Methods:**

Eighty patients undergoing fixed orthodontic treatment with vestibular appliances were enrolled and divided into 2 equal groups: MET for metal brackets and CER for ceramic brackets. After bonding, bracket failures were recorded for 12 months, along with the archwire placed at the time of failure. Angle's dental class, skeletal class, Wits appraisal, Little's irregularity index, overjet, overbite, age, and gender of the patients were recorded from pretreatment cephalometric tracings and study casts. The data were statistically analysed (*P* < .05).

**Results:**

Significantly higher failure rates were found for ceramic brackets in the overall analysis, in the mandibular arch, and in the posterior region. Regression analysis revealed a significant influence of round nickel-titanium archwires on higher failure rates, whilst a significant influence of rectangular archwires was found on lower failure rates.

**Conclusions:**

Ceramic brackets showed higher failure rates. Patients should be aware that orthodontic treatment with ceramic brackets may involve delays and inconvenience due to the higher failure rate compared to metal brackets.

## Introduction

Orthodontic treatment amongst adult patients has become very popular. Recent data indicate, that the percentage of adult American patients receving orthodontisc treatment is around 28%.^1^ Great importance is given to aesthetic appearance, and social concerns can influence the choice of the orthodontic appliance to be used.^2^ Anxiety has been shown to be strongly associated with aesthetic appearance. Reducing anxiety after orthodontic treatment, even in an adolescent context,^3^ justifies continued research into novel designs for orthodontic appliances in the hope of better aesthetic results. Amongst the orthodontic appliances available, ceramic brackets are preferred as a more aesthetic alternative to metallic brackets,^4^ but bracket failure should be considered.

Bracket failure has several implications, such as total treatment time, cost, and patient compliance. Patient motivation, combined with patient age, is certainly an important factor to consider when warning of bracket failure,^5^ considering that up to 46% of the variation in treatment time is due to bracket failure.^6^

Therefore, the goal to date has been to develop orthodontic appliances that combine patient aesthetic requirements with clinical performance to achieve adequate treatment^7^; considering the higher cost of clear aligners and lingual appliances, patients still opt for fixed traditional appliances.^8^

Differences between ceramic and metallic brackets, in addition to their aesthetic appearance, are the greater residual adhesive after debonding—resulting in more difficult removal of resin remnants^9^—and greater bracket–wire friction, involving less efficient dental movements.^10^

Although ceramic brackets are widely used, to the authors’ knowledge, only 2 retrospective studies have clinically compared the failure rates of metallic vs ceramic brackets to date.^11^**^,^**^12^ Therefore, the aim of the present clinical study was to compare the failure rates of metallic and ceramic brackets during the first 12 months of therapy.

The first null hypothesis was that there was no significant difference between the failure rates of metallic and ceramic brackets. The second null hypothesis was that there was no significant difference in the failure rates of orthodontic brackets for upper and lower arches. The third null hypothesis was that there was no significant difference in the failure rates of orthodontic brackets for anterior and posterior sectors.

## Materials and methods

### Study approval

The study was conducted in accordance with the guidelines of the Declaration of Helsinki, and it was approved by the Unit Internal Review Board (No. 2021-0609). The trial was registered on ClinicalTrials.gov (NCT05151991).

### Trial design, participants, eligibility, settings, and consent

The following study is a double-arm, parallel, prospective nonrandomised clinical trial. Patients scheduled to begin orthodontic treatment with fixed appliances with stainless steel or ceramic brackets were enrolled. Patients who participated in the study were selected according to the following inclusion criteria: permanent dentition; completely intact enamel, without traumatic or carious lesions and who had not undergone pretreatment of the tooth surfaces with chemical agents; and patients who had not previously undergone orthodontic treatment with fixed vestibular multibracket orthodontic therapy.

The following exclusion criteria were employed: systemic diseases; medications that could alter periodontal status (eg, nonsteroidal, anti-inflammatory drugs, steroids); absence of congenital enamel defects; absence of dental crowns/bridges; extractions for orthodontic reasons; and patients scheduled for oral or orthognathic surgery.

### Interventions

After patients agreed to begin orthodontic treatment with fixed vestibular appliances, they underwent the direct bonding procedure by a trained operator. After positioning a cheek retractor for the isolation, debris removal was performed with a brush mounted on a low-speed contrangle.^11^ Etching was performed with 37% orthophosphoric acid (Gerhò SpA) applied to the buccal surface of the dental elements for 30 seconds.^13^ The surfaces were rinsed and dried, then a thin layer of Transbond XT Light Cure Adhesive Primer (3M Unitek) was applied and cured with an LED light (Starlight Pro, Mectron SpA) for 10 seconds.^14^ Transbond XT Light Cure Adhesive Paste (3M Unitek) was applied to the base of the metal (Queen Series Low Profile Brackets MBT 0.022", Aestetika Srl) or ceramic (Super Clear Series Brackets MBT 0.022", Aestetika Srl) brackets. The brackets were placed on the buccal surfaces of the teeth, with light pressure sufficient to remove excess cement. At this point, the cement was cured using an LED lamp for 10 seconds per each surface (mesial, distal, gingival, occlusal) of the bracket.^14^ After bonding, orthodontic treatment was performed with the following archwires: 0.012′′ nickel-titanium (NiTi), 0.014′′ NiTi, 0.016′′ NiTi, 0.017′′ × 0.025′′ NiTi, and 0.019′′ × 0.025′′ stainless steel (Orthoshape, Aestetika Srl). NiTi wires were used for levelling and alignment phases, whilst stainless steel wire was used for class mechanics and space closure. Bite-raisings were bonded to all patients on first upper molars when the bonding of the lower arch was performed to avoid early contacts.

### Outcomes

For each dental arch, data collection began at the time of bonding and continued for each patient for the next 12 months. Each failed bracket was recorded once.^14^

Skeletal and dental parameters were collected to perform linear regressions with the failure rates of the 2 study groups. The skeletal measurements were as follows: skeletal class, assessed by Riedel's ANB angle,^15^ and Wits appraisal (A_0_B_0_).^16^ Measurements were made on pretreatment lateral cephalometric radiographs. The following dental measurements were carried out on pretreatment study casts: Angle's dental class, Little's irregularity index (LI),^17^ space analysis (SA),^18^ overbite, and overjet. Measurements were performed with a dental caliper (Leone SpA).

Two calibrated operators not involved in clinical procedures performed the cephalometric tracings and the measures on the dental casts. To assess interrater and intrarater reliability, 10 patients were randomly selected; their measurements were repeated after 14 days and intraclass correlation coefficient (ICC) and Cohen's kappa (κ) were respectively calculated.

### Sample size

The sample size was calculated based on the percentage of bracket failures from the results of the work of Kirschneck et al.^19^ The power to detect a clinically relevant difference of 4.935% in the percentage of failures was based on an expected value of 12.08. Setting a Type I alpha error of 0.05 and a Type II beta error of 85%, 800 brackets and, therefore, 40 patients in each group were required.

### Randomisation and blinding

The randomisation process was not possible due to the design of the study. The operators who performed skeletal and dental measurements were blinded to which group (metallic or ceramic brackets) patients were in. The data analyst was blinded for data analysis.

### Statistical methods

Data were statistically analysed using R software (R version 3.1.3, R Development Core Team, R Foundation for Statistical Computing) by calculating mean and standard deviation as descriptive statistics. Fisher exact test was performed to evaluate the differences between the failure rates of the 2 groups tested. Kaplan–Meier survival curves were calculated with a log-rank test to examine the failures and the time frame in which they occurred. Finally, linear regressions were performed to evaluate the effect of the different study variables on bracket failures. For all tests, significance was set at *P* < .05.

## Results

### Participant flow and baseline data

The study began in December 2021 and ended in June 2023. Eighty patients were included in the study, 40 in the metal (MET) group (mean age, 24.36 ± 13.48 years) and 40 in the ceramic (CER) group (30.73 ± 11.41 years). Descriptive statistics of the study variables (mean, standard deviation, minimum, maximum, and median) are shown in Table 1.

**Table 1:** Descriptive statistics of the variables of the study.

Group	Age (y)	ANB (°)	A_0_B_0_ (mm)	OB (mm)	OJ (mm)	LI (mm)	SA (mm)
MET	24.36 ± 13.48	2.77 ± 2.10	1.28 ± 3.71	3.72 ± 1.92	3.53 ± 1.71	4.89 ± 2.47	−0.63 ± 3.26
CER	30.73 ± 11.41	4.09 ± 2.78	2.98 ± 4.53	3.99 ± 2.09	3.61 ± 2.31	4.27 ± 3.01	−1.68 ± 3.86

Values are mean ± standard deviation.

MET, metallic brackets; CER, ceramic brackets; ANB, Riedel's ANB angle; A_0_B_0_, Wits appraisal; LI, Little's irregularity index; SA, space analysis.

**Fig. 1:**
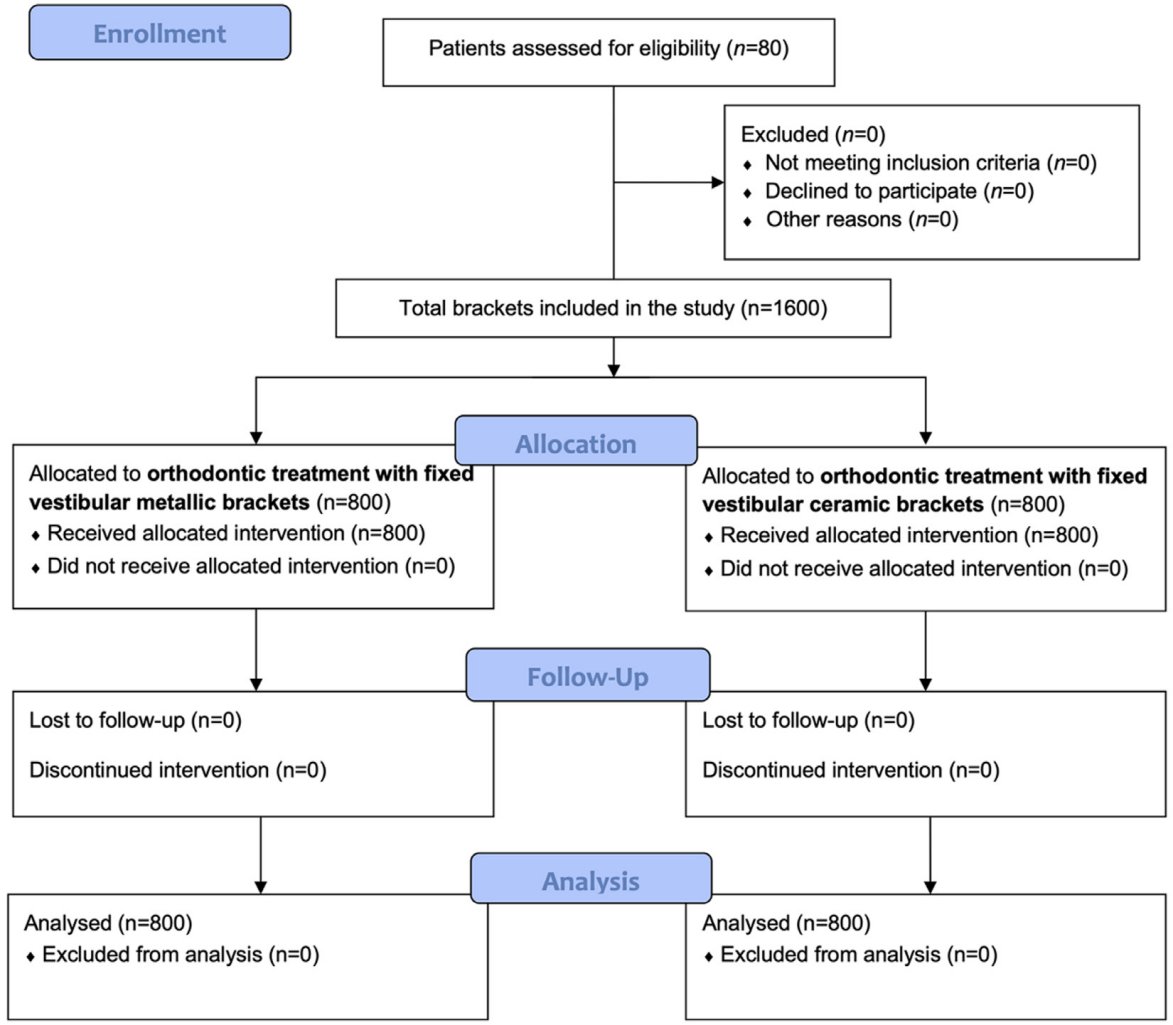
CONSORT flowchart of the study showing the phases of enrolment and analysis.

Intrarater reliability resulted in almost perfect agreement (ICC = 0.985), just as interrater reliability (κ = 0.969). The flowchart of the study is shown in Figure 1.

### Numbers analysed for each outcome, estimation and precision, subgroup analyses

The failure rate of ceramic brackets compared to metal brackets, considered over the first 12 months of therapy and without distinction of sector (anterior/posterior) or arch (upper/lower) is higher and statistically significant in the CER group (*P* < .0001; Table 2).

**Table 2:** Fisher exact test for the comparison of total failure rate.

Group	No. bonded	No. failures	Failure rate (%)	Significance*
MET	800	73	9.13	
CER	800	127	15.88	.0001*
Total	1600	200	12.50	

⁎ Significance threshold set for *P* < .05. MET, metallic brackets; CER, ceramic brackets.

**Fig. 2:**
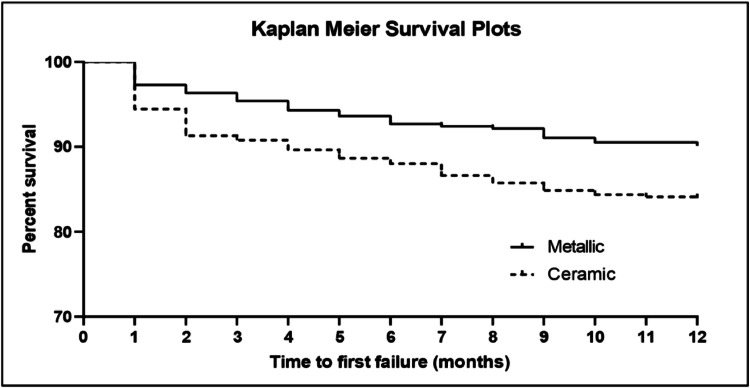
Kaplan–Meier survival curves for the 12 months of treatment.

Kaplan–Meier curves are shown in Figure 2. Statistically significant differences were found in terms of the risk of failure between the 2 groups tested during the 12 months of the trial (hazard ratio, 0.54; 95% confidence interval, 0.41–0.72; log-rank test, *P* = .0002).

In the sector analysis (Table 3), a significantly higher failure rate was found for ceramic brackets in the posterior sector (*P* < .05). However, no significant difference was found in the anterior sector (*P* > .05). When comparing the overall failure rate of anterior and posterior sectors, a significantly higher failure rate was found for the posterior sector (*P* < .05).

**Table 3:** Fisher exact test for the comparison of the failure rates for anterior and posterior brackets.

Group	Anterior			Posterior			*P* value*
	No. bonded	No. failures	Failure rate (%)	No. bonded	No. failures	Failure rate (%)	
MET	480	46	9.58	320	27	8.44	
CER	480	45	9.38	320	82	25.63	
							
Total	960	91	9.48	640	109	17.03	.0001*
Intragroup *P* value			.9796			.0001*	

⁎ Significance threshold set for *P* < .05. MET, metallic brackets; CER, ceramic brackets.

When analysing the dental arches (Table 4), a significantly higher failure rate for ceramic brackets was found in the lower arch (*P* < .05), whilst no significant difference was found in the upper arch (*P* > .05). When comparing the arches, the lower arch had a significantly higher failure rate (*P* < .05).

**Table 4:** Fisher exact test for the comparison of the failure rates for upper and lower arches.

Group	Upper arch			Lower arch			*P* value*
	No. bonded	No. failures	Failure rate (%)	No. bonded	No. failures	Failure rate (%)	
MET	400	30	7.5	400	43	10.75	
CER	400	42	10.5	400	85	21.25	
							
Total	800	72	9	800	128	16	.0001*
Intragroup *P* value			.1208			.0001*	

⁎ Significance threshold set for *P* < .05. MET, metallic brackets; CER, ceramic brackets.

Regarding the linear regressions, a significant effect (*P* < .05) of group, NiTi round archwires 0.014” and 0.016”, round archwires, and rectangular archwires on bracket failure was found (Table 5).

**Table 5:** Linear regressions of the variables of the study.

	Failure rate
Independent variable	*P* value
Sex	.220
Age	.072
Group (MET/CER)	.046*
ANB	.356
A_0_B_0_	.077
Skeletal class	.929
Left molar class	.282
Right molar class	.349
Left canine class	.493
Right canine class	.233
Overbite	.987
Overjet	.237
Little's index	.127
SA	.698
00.12” NiTi	.179
00.14” NiTi	.0001*
00.16” NiTi	.038*
0.017” × 0.025” NiTi	.093
0.019” × 0.025” SS	.349
Round archwire	.0001*
Rectangular archwire	.004*

⁎ Significance threshold set for *P* < .05. ANB, Riedel's ANB angle; A_0_B_0_, Wits appraisal; LI, Little's irregularity index; SA, space analysis; NiTi: nickel-titanium; SS, stainless steel; MET, metallic brackets; CER, ceramic brackets.

## Discussion

### Main findings in the context of the existing evidence and interpretation

A significant number of patients perceive commonly used orthodontic appliances as unattractive and unacceptable, preferring clear aligners and lingual braces to ceramic and metallic vestibular brackets.^20^ As orthodontic treatment with vestibular brackets is a popular choice of adult patients,^1^^,^^21^ it is important to study the pehnomenon of bracket failure. This issue is interesting yet complex, due to the varying causes and variables that are involved, the most discussed topics in this context are the bonding techniques and adhesive materials used in bracket fixation.^22^**^,^**^23^ Indeed there are a number oif contemporary reviews on this usbject in the international literature including systematic reviews and meta-analyses.^22^**^,^**^24^

Regarding materials properties, in vitro studies focused on the bracket–resin and composite–adhesive–substrate interfaces, which should be sufficiently strong to prevent bracket failure in the presence of orthodontic and masticatory forces but not too strong to allow for easy debonding at the end of therapy.^25^ Bonding forces should not exceed 40 to 50 MPa, as above this threshold there is a high probability of enamel loss during debonding.^26^**^,^**^27^ On the other hand, the minimum bond strength to withstand masticatory forces is 5 to 10 MPa.^28^ In clinical practice, a systematic review reports that the incidence of bracket failure can vary within a wide range of 0.8% to 28.3%^29^; however, attempts should be made to keep the rate as low as possible. Different factors can influence bracket adhesion and, therefore, bracket failure: age and sex,^30^ treatment duration,^31^ enamel pretreatment procedures,^14^ enamel contamination before bonding,^32^ precoating of brackets,^13^ conditioning agent,^33^ adhesive system,^34^ curing light,^35^ and its power output.^36^ As no study has focused on the clinical performances of vestibular metallic and ceramics brackets in the same setting, the objective of the present nonrandomised clinical trial was to compare the failure rates of both types of brackets in a 12-month study.

The first null hypothesis of the present study was rejected. In fact, in the overall analysis statistically significantly more failures were found for ceramic brackets. The failure rates of both the types of brackets were in line with the range in previous literature,^29^ with a higher rate for ceramic brackets.^13^**^,^**^24^ However, no direct comparison had been previously performed between the 2 types of brackets, but other variables were evaluated, including enamel demineralisation, bonding time, adhesive removal time, adhesive remnant index, and precoating of the base of the brackets. The finding of the present study is confirmed by Stasinopoulos et al,^12^ whilst is in contrast with the study by Ogiński et al,^11^ which found a significantly higher failure rate for the metallic brackets. However, they were both retrospective studies with a small sample. With regards to in vitro studies, however, it should be noted that conflicting results have been reported; in fact, ceramic brackets have higher^37^, ^38^ or lower^39^^,^^40^ shear bond strength (SBS) values compared to metallic brackets. Laboratory research is useful for preliminary evaluations, but its findings should be interpreted with caution because different results can be found when the same experiments are translated to the clinical setting; interestingly, lower SBS was found when brackets were debonded in patients compared to in vitro extracted premolars.^41^

The fact that different SBS values have been found for ceramic brackets could be due to several factors. For example, the bracket base may have various dimensional differences between metal and ceramic brackets, but this does not seem to be related to SBS, as suggested by data from previous studies.^37^**^,^**^39^**^,^**^42^ Nevertheless, it should be noted that factors such as enamel contamination and light curing during the bonding procedure,^35^**^,^**^36^ which are not specified in some studies, should be considered in clinical setting.

In addition, the risk of fracture of ceramic brackets should be analysed, taking into account the potential loss of enamel to which they are bonded.^43^

The second and the third null hypotheses were partially rejected, as a significantly higher failure rate was found for ceramic brackets in the posterior sector and in the lower arch, whereas no difference between the 2 techniques was detected in the anterior sector or in the upper arch. Several studies in the literature report a higher incidence of failures in the posterior and lower sectors for metallic brackets,^44^**^,^**^45^ whilst for the ceramic brackets the difference is less marked.^24^ The explanation may be related to greater masticatory forces on posterior regions with respect to incisors.^41^ However, as this study did not include molar tubes, future studies that do should be performed to completely confirm this.

Regarding skeletal and dental parameters for the regression analysis, previous studies assessed the influence of younger age,^30^**^,^**^44^ increased overbite,^44^^,^^46^ and early stages of treatment.^13^**^,^**^31^**^,^**^44^ In the present study, a significant influence of bracket material was found, confirming the results of Fisher exact test in favour of metallic brackets in terms of bracket failure. Moreover, significantly higher failure rates were found for 0.014” and 0.016” NiTi archwires from regression analysis, just like considering round archwires in toto. These results are consistent with previous studies that found higher failure rates in the first few months.^11^**^,^**^31^**^,^**^44^ Conversely, rectangular archwires had a significant impact on bracket failure, as the failure rate was significantly lower at the end of the study. The fact that bracket failure rates are higher in the early stages of orthodontic treatment could be due to several factors, as follows: patients may have difficulties chewing properly whilst wearing orthodontic appliances, leading to a lack of attention and preventing bracket displacement. Additionally, round NiTi archwires can cause tooth displacement during initial levelling and alignment movements. As the linear regression of Little's irregularity index was not significant, and the data does not support the possibility that dental crowding could be a cause of bracket failure; in fact, from this study, bracket failure rates were not significantly higher in the anterior region, although the anterior mandibular region is known to be affected by dental crowding.^46^ Even though a previous study assessed that moderate crowding could be significantly involved in higher bracket failure rates,^47^ it should be noted that the age range of the considered study is older, and dental crowding is known to directly increase with age.^48^ In addition, space analysis was considered, but the related linear regression results were not significant.

### Limitations

The present study has some limitations: the age of sample was relatively young. Considerations on age-related factors with a stratification for older age ranges could be performed in future randomised clinical trials, including patients from the same limited age range. The same experimentation should be performed also amongst other study populations and with different types of brackets and/or adhesive materials. The influence of bite-raising on bracket failure should be evaluated via controlled studies. Patients’ compliance amongst the groups could not be evaluated, as the present study is a nonrandomised trial, but future investigations could include evaluation of compliance.^49^ Finally, further studies could be designed to evaluate other factors such as total treatment time, total number of appointments, costs, and how these variables are related to bracket failures, in addition to compliance-related factors.

## Conclusions

In general, we noted significant higher failure rates of ceramic brackets compared with metal brackets particularly for lower arch and posterior sector. Significantly more bracket failures were found in the first 12 months when using round wires, highlighting the fact that failures mainly occur during the first month of treatment. Patients should be aware of this eventuality when choosing the material of their vestibular fixed appliance in orthodontic treatment.

## CRediT authorship contribution statement

**Andrea Scribante:** Conceptualization, Methodology, Software, Formal analysis, Validation, Writing – review & editing. **Maurizio Pascadopoli:** Data curation, Writing – original draft. **Paola Gandini:** Resources, Supervision, Project administration, Funding acquisition. **Riccardo Mangia:** Investigation. **Costanza Spina:** Investigation. **Maria Francesca Sfondrini:** Conceptualization, Methodology, Validation, Writing – review & editing.
